# New Appliances and Things Medical

**Published:** 1901-07-06

**Authors:** 


### NEW APPLIANCES AND THINGS MEDICAL.

[We shall be glad to receive, at our Office, 28 & 29 Southampton Street, Strand, London, W.C., from the manufacturers, specimens of all new preparations and appliances which may be brought out from time to time.]

### IDEAL MILK.

(Anglo-Swiss Condensed Milk Company, 10 Mark Lane, London, W.C.)

We have received a sample of "Ideal Milk" which is manufactured by the same firm as the well-known "Milk Maid" brand. It possesses certain very solid advantages over other varieties of condensed milk, and for the following reasons: Firstly, it contains no added sugar; secondly, it is enriched by a considerable proportion of added cream; and thirdly, it has been submitted to a complete process of sterilisation. In those cases in which through necessity or choice an infant is reared on condensed milk "Ideal Milk" may well be employed to the exclusion of other varieties. The disastrous consequences of feeding infants on ordinary brands of condensed milk are due mainly to the following causes: 1. Poisoning by enormous excess of added sugar; 2. Deficiency of fat. As far as these points are concerned the employment of "Ideal Milk" is to obviate them. Further than this "Ideal Milk" is exceedingly palatable, not only to infants but also to adults. For tea, coffee, cocoa, &c., for puddings, or for ordinary drinking purposes it is as different from ordinary condensed milk as curds are from cheese, and we strongly recommend readers of The Hospital to give it a trial.

### PLASMON COCOA.

(International Plasmon, Limited, 56 Duke Street, Grosvenor Square, W.)

There can be no doubt that this new departure in the preparation of cocoa has very decided advantages from the point of view of general nutrition. Until recently it has been a cherished belief that sugars and starches were the simplest and best forms of nutritive material for growing children and for invalids. As a matter of fact, those who have paid most attention to the subject of dietetics recognise that restriction of starches and sugars is often absolutely necessary, and a liberal supply of simple proteid food is the proper food substitute for such individuals. Plasmon cocoa is an ideal food in this respect, for the nutritive properties of the cocoa are supplemented by the addition of the best of all proteids, namely, milk proteid. For this reason we strongly recommend to nurses and mothers this variety of cocoa, more especially as a substitute for those forms of the beverage which are thickened with starch or flour, and have therefore enjoyed a most undeserved reputation as a form of nutriment for growing children. The majority of infantile complaints, as well as of those to which adults are subject, are often directly due to the excessive ingestion of these same articles of food which tradition has caused to be regarded as quite harmless. Sugar and starches are essential for those whose occupation necessitates the display of much muscular energy; excepting in very moderate quantities they are not really necessary, but actually harmful, for the majority of persons living under the artificial condition of civilisation; Plasmon cocoa is a proper diet if we wish our children to be strong and healthy as opposed to being fat and liable to disease.

### VACCINATION SHIELDS.

(Wm. Cowan, 48 & 50 Dundas Street, Glasgow.)

These shields are simple and effective; they consist of a wide meshed wire-netting moulded to the shape of the arm, and protected round the edges where in contact with the skin by a well-designed wash-leather pad. The little apparatus is held in position by silk bands which are loosely tied round the arm. If properly adjusted, the points of inoculation are absolutely protected from friction or blows, and at the same time are in full communication with the air. They are well designed for the purpose of protecting the scabs and for ensuring rapid healing.

